# Compressive Optical Image Encryption

**DOI:** 10.1038/srep10374

**Published:** 2015-05-20

**Authors:** Jun Li, Jiao Sheng Li, Yang Yang Pan, Rong Li

**Affiliations:** 1 Guangdong Provincial Key Laboratory of Quantum Engineering and Quantum Materials, School of Physics and Telecommunication Engineering, South China Normal University, Guangzhou 510006, China

## Abstract

An optical image encryption technique based on compressive sensing using fully optical means has been proposed. An object image is first encrypted to a white-sense stationary noise pattern using a double random phase encoding (DRPE) method in a Mach-Zehnder interferometer. Then, the encrypted image is highly compressed to a signal using single-pixel compressive holographic imaging in the optical domain. At the receiving terminal, the encrypted image is reconstructed well via compressive sensing theory, and the original image can be decrypted with three reconstructed holograms and the correct keys. The numerical simulations show that the method is effective and suitable for optical image security transmission in future all-optical networks because of the ability of completely optical implementation and substantially smaller hologram data volume.

With the increased importance of information security, image security has become increasingly important in many current application areas^1^. The study of image security includes image encryption, image hiding and image watermarking^2^,^3^,^4^,^5^. Image encryption technology has been widely applied to many application areas, such as 3D image encryption, data monitoring, data tracking and confidential data transmission in the military and medical fields^6^, quantum-secured imaging^7^, and quantum-secured surveillance^8^. In recent years, image security that fully utilises optical parallel features has become an important research topic^9^,^10^,^11^; we also have demonstrated the feasibility of optical image hiding and optical image encryption and hiding^12^,^13^ These methods may be effective solutions to the future implementation of all-optical systems^14^.

However, the large volume of data required for storing or transmitting holograms has been a main limiting factor of optical image security. Many hologram compression schemes have been reported in recent years to solve this problem^15^,^16^; however, their effectiveness is limited by the introduction of hologram laser speckling^17^, and the realisation of hologram compression is typically performed using electronic means. The newly developed theory of compressive sensing (CS)^18^,^19^,^20^,^21^ provides a new technical approach for hologram compression in the optical domain^22^,^23^,^24^ and captures the non-adaptive linear projections of compressible signals at a rate that is significantly below the Nyquist rate. These signals are then reconstructed from these projections using an optimisation process. Simultaneously, CS is combined with other special imaging methods to obtain wider application, such as in quantum imaging^25^,^26^, photon counting imaging^27^, the coherent imaging of different wavelengths^28^, and the measurement of electric fields^29^,^30^. These features may also be effective solutions for the massive data processing and information security requirements of the Internet of Things (IoT)^31^. Recently, various image encryption methods based on compressive sensing, such as parallel image encryption^32^, image encryption with an Arnold transform^33^,^34^,^35^ and colour image encryption^36^, have been proposed. However, these methods relate to digital image encryption; completely optical schemes for image encryption based on compressive sensing have not been discussed.

This paper proposes a completely optical image encryption method based on compressive sensing. Using a Mach-Zehnder interferometer, an object image is first encrypted to a white-sense stationary noise pattern using a DRPE^37^ method in the object beam path. Then, the encrypted image is highly compressed to a signal utilising the sparsity of the signal in a sparse domain. At the receiving terminal, the encrypted image reconstruction is achieved from small amounts of data by an optimisation process, and the original image can be decrypted with three reconstructed holograms and the correct keys. The method can be used to perform compressive optical image encryption in a purely optical system; therefore, it is effective and suitable for secure optical image transmission in future all-optical networks. Moreover, our method utilises the sparsity of a signal to reconstruct a complete signal from a small sample to overcome the limitation of the large hologram data volumes of 3D images or 3DTV. In addition, our method can overcome the limitations of the precision and costs of traditional sensors, wavelengths and resolution for array imaging based on CS. The principles and numerical simulations are described below.

## Methods

**Figure 1:**
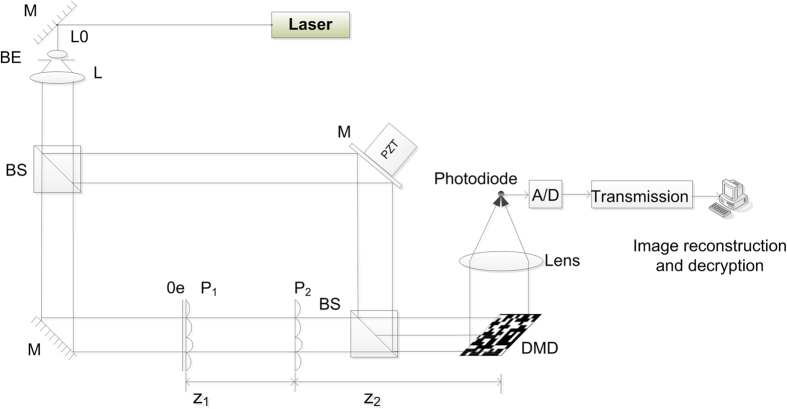
Setup of compressive optical image encryption. BE, beam expander; L, lens; BS, beam splitter; M, mirror; P, random phase plate; PZT, piezoelectric transducer mirror; Oe, object image.

The compressive optical image encryption system is shown in Fig. 1. A laser beam is split into an object beam and a reference beam. The object beam first illuminates an object image that is used for encryption and subsequently passes through two random phase masks 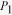 and 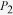 to perform the encryption using the DRPE method. In the other arm, the reference beam illuminates the piezoelectric transducer mirror (PZT), which is capable of phase shifting. Then, the two waves overlap to form an interference pattern in the plane of a Digital Micromirror Device (DMD). The DMD, a semiconductor-based “light switch” array of millions of individually addressable, tiltable mirror-pixels, is a reflective spatial light modulator. Each tiltable mirror-pixel can be rotated +12 or −12 degrees from the horizontal to reflect light to or away from an intended target. When the mirror-pixel is in the +12 degree state, more than 88% of the reflected energy can be coupled to the target^38^. Then, the compressive sampling data are obtained by the photodiode detector with the modulation of the encrypted complex light field by the DMD device. Finally, we can acquire the compressed hologram image by a traditional communication channel and subsequently reconstruct it via the specific algorithm. In addition, the original object can be decrypted via an inverse Fresnel transformation with three reconstructed holograms and the correct keys.

Let us assume that the reference wave is simply given by the real amplitude 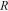, and a complex object field in the plane 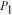 is 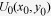. The complex amplitude transmittances of the random phase plates 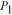 and 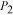 are 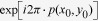 and 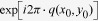, respectively, where 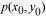 and 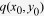 are two independent white noises uniformly distributed in[0, 1]. The distance between plates 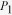 and 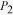 is 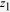, and that between 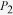 and DMD is 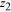. The complex object field 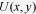 on the DMD plane can be described as
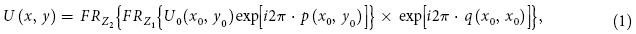
where 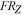 denotes the Fresnel transform of the distance 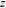. Based on traditional three-step, phase-shifting holography, the phases of the reference wave can be modulated by PZT to 0, 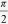 and 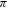. Then, the three interference intensities of the complex amplitude field on the DMD plane are expressed as
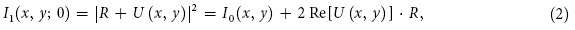

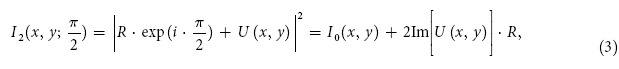

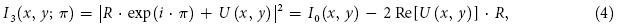
where 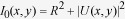 When the complex amplitude field on the DMD plane is modulated by the DMD device and coupled to the photodiode detector via a lens, we can obtain compressive sampling data of the three encrypted interference patterns on the photodiode. Specifically, the final output value on the photodiode detector can be calculated by computing the inner product of the intensity value 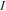 of the complex amplitude field and the measurement matrix 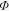 loaded into the DMD device. When we repeat the process 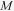 times, we can obtain the full measurements 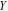 of the compressive sampling. For example, we obtain the measurement data 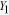 through 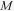 computations of random linear measurements of intensity 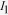 and the measurement matrix 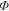 in the DMD device. The processing can be expressed as follows:
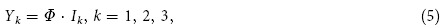
where 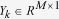 is the measurement data obtained in the single-pixel detector, 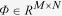 is the measurement matrix generated by the DMD, 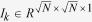, 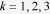, “

” means the inner product operation.

Then, we transmit the measurement data and measurement matrix using a conventional channel to the computer, where the image reconstruction and decryption will be performed. For the image signal, because the gradients of most images are sparse, Rudin *et al.* presented a nonlinear total variation (TV) algorithm, which attempts to denoise images in an effective manner. Simultaneously, it can enforce a sparsity constraint and reconstruct images well under compressive sensing theory^39^. The concept of constrained TV minimisation, which attempts to minimise the gradients of images, originated from the field of compressive sensing in the work by Candes *et al.*^18^ Specifically,
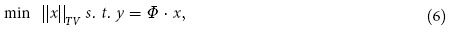
where
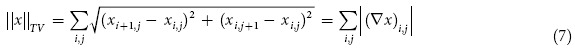
is the total variation in the image x: the sum of the magnitudes of the gradient. The image can be constructed by solving the convex optimisation problem of minimising the 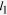-norm of the image subject to the constraint that the image’s DFT matches the measured DFT values. The null space property (NSP) is a sufficient condition for 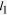 convex minimisation to obtain the sparsest solution. We are primarily concerned with how well CS can approximate a given signal from a given budget of fixed linear measurements compared to adaptive linear measurements^40^. Therefore, the advantages of using total variation (TV) is that the TV can considerably reduce the under-sampling ratio as well as offer robustness to noise in the data due to the better null space property (NSP). In the traditional approach of using a regulariser, such as TV, there is a trade-off between data fidelity and image regularity. A group at Duke also developed a TV algorithm to reduce the noise of a compressed hologram and solve the linear inversion problems^41^. In the present work, we are interested in image reconstruction in which the measurement is incomplete. Because of the incompleteness, there will be no unique minimiser of the data-fidelity-objective function, and TV is used to select a unique image out of the set of possible images that agree with the available data.

We first adopt two-step iterative shrinkage (TwIST) algorithm to reconstruct the interference wave intensity 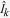 by solving the optimal problem under additive white Gaussian noise in the system.
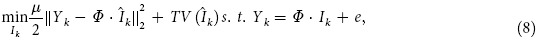
where 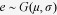 is measurement noise, 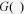 is the Gaussian noise, 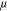 is the mean value, 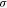 is the standard deviation, 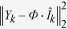 is the 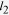 norm of 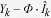, and 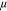 is a constant. The first penalty is a least-squares term that is small when 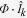 is consistent with the correlation vector 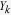. The second penalty 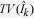 is the signal’s total variation. As a result, a solution to the ill-posed equation that satisfies both the minimum error energy and minimisation of the recovered signal’s total variation is obtained. From the above theoretical analysis, the interference wave intensity 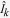 can be correctly computed by solving the convex optimisation only when the receiver obtains the correct measurement matrix. This proves that the system’s security is improved with the addition key space of the measurement matrix; the merits of the system are further discussed in the numerical simulation in the next section.

When the intensity patterns 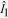, 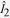, 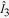 are reconstructed with the TwIST algorithm, we can calculate the complex amplitude on the DMD plane
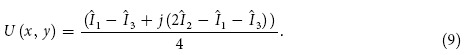


Once the complex amplitude 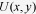 is known, in addition to random phase masks 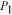 and 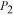, *z*_1_, *z*_2_ and λ, we can digitally or optically retrieve the original object image from the encrypted image as
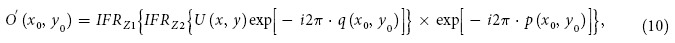
where 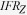 denotes the inverse Fresnel transformation of the distance 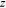. Specifically, the original image has been perfectly reconstructed and decrypted.

## Results

**Figure 2:**
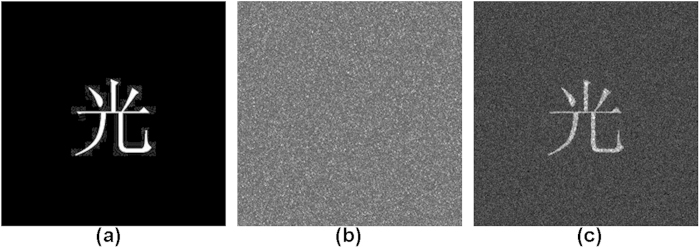
Simulation results with a binary image. (**a**) Binary image; (**b**) one of the encrypted holograms on the DMD plane; (**c**) retrieved image from 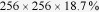 measurements using our method.

**Figure 3:**
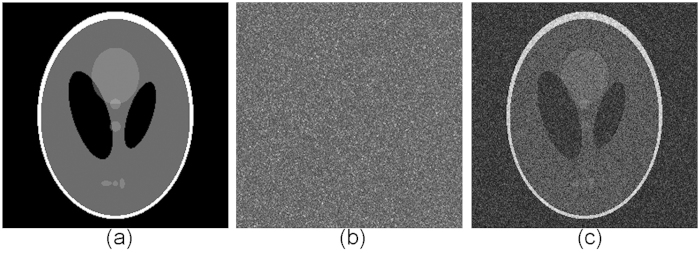
Simulation results with a gray-level image. (**a**) Gray-level image; (**b**) one of the encrypted holograms on the DMD plane; (**c**) retrieved image from 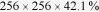 measurements using our method.

A series of simulations have been performed to verify the feasibility of our proposed method. This section presents a series of results based on the following conditions. The type of central processing unit (CPU) used in the computer simulation is an Intel(R) Core(TM) i7, and the memory of the computer is 6 GB. We used the MATLAB R2009a software package. The parameters that we used were 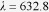
nm, 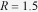, and 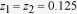 *m*. The measurement matrix size of the DMD used in the computer simulation was 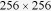 pixels. The measurement matrix generated by the DMD is random sequences of 0/1. The original object images used in the simulation are shown in Figs. 2(a) and 3(a), all with sizes of 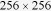 pixels. The intensity values of the complex amplitude field containing encrypted object information are first modulated by the DMD, and then, once we received the compressed data of the encrypted image in the photodiode detector, we can reconstruct the original image from the compressed and encrypted image using the correct keys and the optical system’s parameters. The simulation results for the compressive optical image encryption in a Mach-Zehnder interferometer are shown in Figs. 2 and 3. After performing compressive optical image encryption on the object images, the three encrypted interferograms containing the secret image information on the DMD plane will be sampled with compressive sensing theory; one of these interferograms for Figs. 2(a) and 3(a) is shown in Figs. 2(b) and 3(b). Figures 2(c) and 3(c) show the recovered object images from 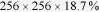 measurements and 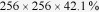 measurements using our method. The computer simulations show that this compressive optical image encryption method is performed using a completely optical scheme in the Fresnel domain.

**Figure 4:**
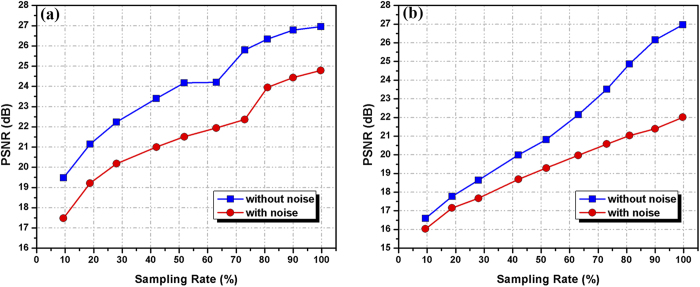
Relations of the sampling rate and PSNR with measurement noise and without measurement noise. (**a**) Relations of the sampling rate and PSNR between the original binary image and reconstructed images; (**b**) Relations of the sampling rate and PSNR between the gray-level image and reconstructed images.

**Figure 5:**
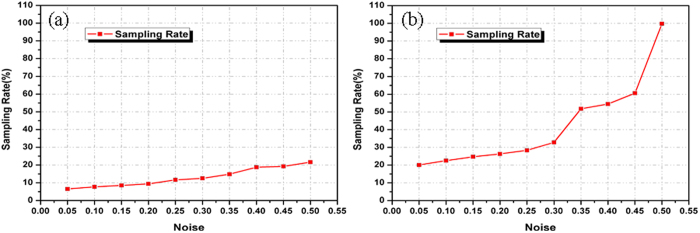
Relations of the number of measurements and the standard deviation 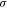 of the measurement noise when the PSNR is 18 dB with (**a**) a binary image and (**b**) a gray-level image.

Moreover, we investigated the compression feature and the effects of the measurement noises in our method. The computer simulations are shown in Figs. 4 and 5. The relations of the sampling rate in the compressive sampling process and the peak signal-to-noise ratio (PSNR) between the original image and the reconstructions are shown in Fig. 4. In this simulation, the measurement noise described in Equation (8) is additive white Gaussian noise with a zero mean and a standard deviation 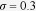. Figures 4(a) and 4(b) present the relations of the sampling rate in the compressive sampling process and the PSNR for a binary image and gray-level image. The PSNRs clearly increase with increases in the sampling rate. When the sampling rate reaches 20%, the PSNR values are close to or greater than 20 dB. When considering the effects of white Gaussian noise, the relations of the sampling rate and PSNR have the same tendency. Figure 5 also illustrates the minimum number of measurements that enables a decent image retrieval under different measurement noise conditions. Figures 5(a) and 5(b) show the relations of the number of measurements and the standard deviation 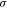 of the additive white Gaussian noise when the PSNR is 18 dB for the binary image and the gray-level image. This shows that the proposed method is effective and exhibits a good performance with a white Gaussian noise in the fully optical domain.

**Figure 6:**
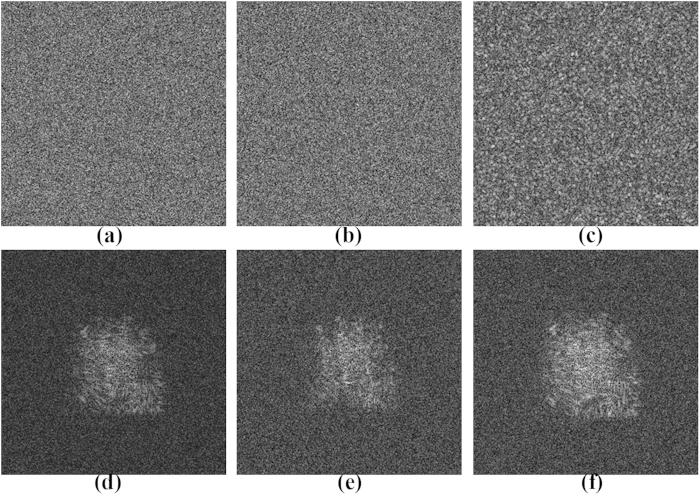
Retrieved images with one incorrect key in the decryption process: (**a**) When the principal key 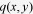 is not used; (**b**) when the measurement matrix 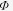 is incorrect; (**c**) when only 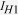 is used; (**d**) when 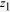 has a relative error of 1%; (**e**) when 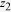 has a relative error of 1%; (**f**) when 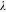 has a relative error of 3%.

To investigate the security of this compressive optical image encryption method, we reconstruct the original binary image when one of the keys is incorrect, as shown in Fig. 6. Once one of the keys is incorrect, the retrieved image will be greatly affected. Among these keys, the principal key 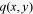 and measurement matrix play critical roles in the security system, as shown in Figs. 6(a) and 6(b), in which the retrieved image is the same as the noise and fully unrecognisable when they do not be used to the reconstruction; when only one compressive sampled hologram dataset is used, there is no correct reconstruction image, as shown in Fig. 6(c); when the two diffraction distances of the object image and the wavelength of the laser exhibit a relative error, there are no correct reconstruction images, as shown in Figs. 6(d)-(f). We have also performed some simulations with other gray-level images and obtained satisfactory retrieval results, which are similar to those obtained with the binary images. Therefore, they are not considered here.

**Figure 7:**
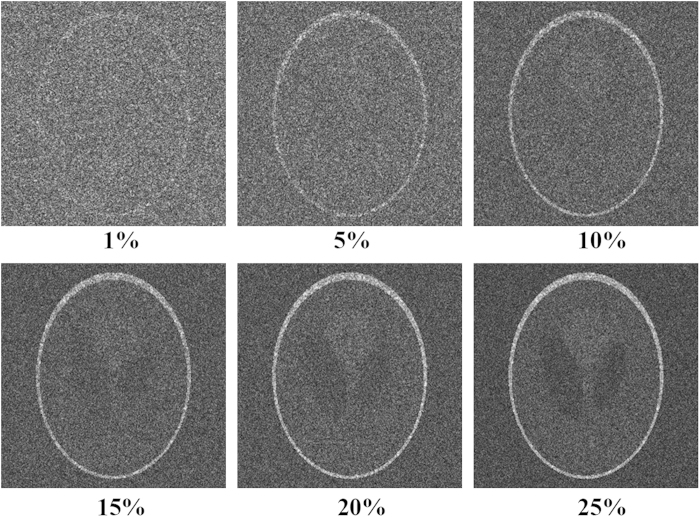
Reconstructed images for partially recovered keys.

**Figure 8:**
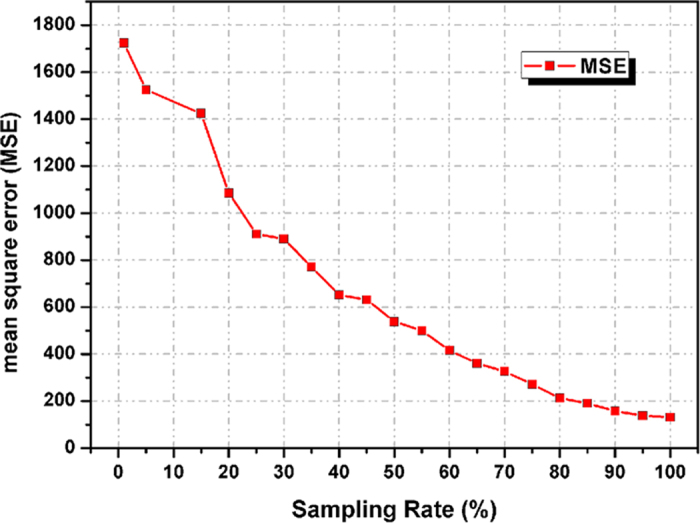
Mean square error (MSE) between the original image and reconstructions versus the sampling rate.

To further test the vulnerability of the proposed compressive encryption scheme, let us assume that a potential eavesdropper, who knows our reconstruction mechanism, has unauthorised access to a fraction 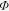 of the key parameters and uses the corresponding measurement matrix to reconstruct the image. Figure 7 shows a sequence of object images reconstructed from such a partially recovered key. The encrypted information begins to be retrieved when 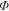 is as high as 15%, which means that the eavesdropper should capture at least 15% of the measurement matrix numbers. To ensure security against more sophisticated eavesdropping attacks, Alice and Bob might want to synchronously and randomly alter the order of the elements of the key for different objects. The mean square error (MSE) between the original image and reconstructions versus the sampling rate is shown in Fig. 8. Clearly, the MSEs are observed to decrease with increases in the sampling rate. This proves that in addition to the keys above, the data of the measurement matrix are also one of the important keys. Therefore, the space of the key is expanded.

**Figure 9:**
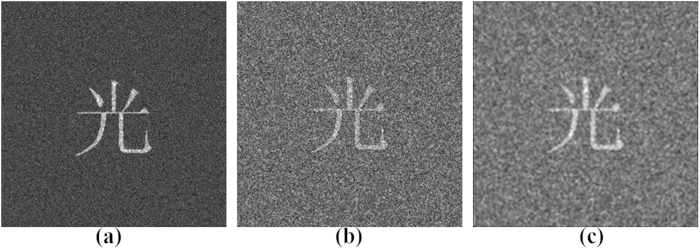
Study of binarisation of the reconstructions. (**a**) Recovered object image from 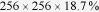 measurements; (**b**) recovered object image is binarised and (**c**) (**b**) filtered by an average filtering with a 3×3 window size.

**Figure 10:**
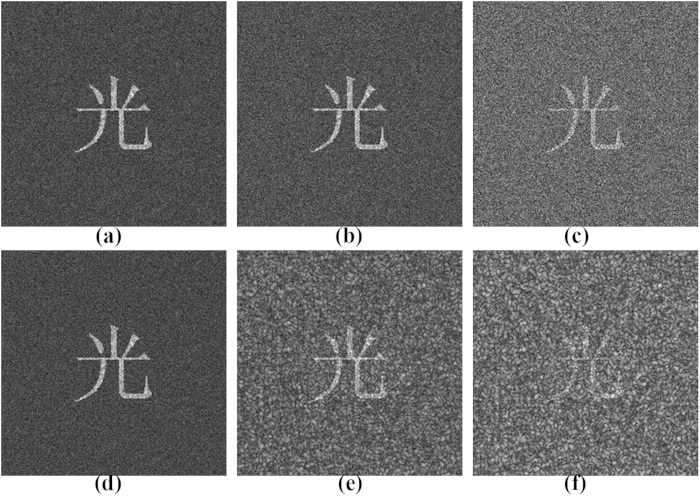
Study of noise perturbations in the encrypted image. The recovered object image from 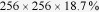 measurements is corrupted by additive white Gaussian noise with the standard deviation (**a**) 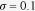; (**b**) 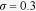; (**c**) 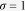; multiplicative noise with the standard deviation (**d**) 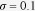; (**e**) 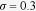; (**f**) 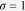.

**Figure 11:**
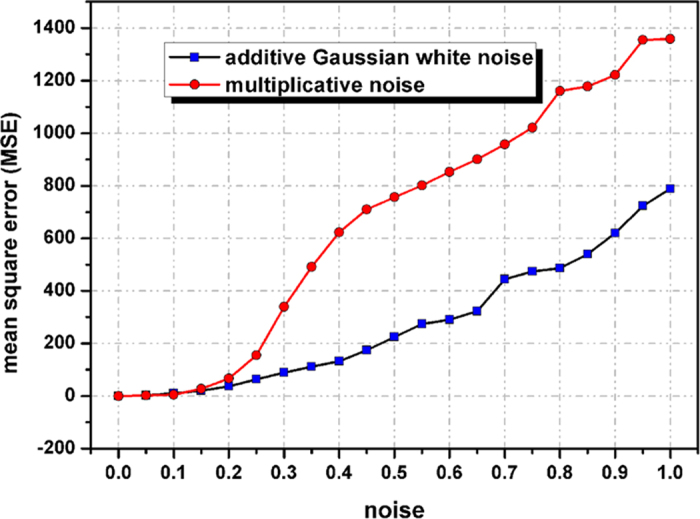
Mean square error (MSE) between the original image and reconstructions versus the standard deviation 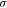 of the additive white Gaussian noise and multiplicative noise.

In addition, a series of simulations have been performed to investigate the robustness of our proposed method. In this section, we study the influence of the binarisation of the encrypted image on the recovered image and evaluate the noise performance of our proposed method in the presence of additive white Gaussian noise and multiplicative noise. Figures 9(a) and 9(b) show a recovered object image from 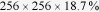 measurements and a recovered object image that has been binarised, respectively, where an average filtering with a 3×3 window size is applied in the binarisation image, as shown in Fig. 9(c). Figure 10 shows the noise perturbation on the recovered object image with different standard deviations of additive white Gaussian noise and multiplicative noise. We calculated the MSE between the original and noise-corrupted recovered object images after using all of the correct keys. Figure 11 shows the MSE between the original image and reconstructions versus the standard deviation 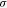 of the additive white Gaussian noise and multiplicative noise. The MSEs decrease as the standard deviations 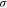 increase, and the multiplicative noise has a greater influence on the recovered object image compared with additive white Gaussian noise.

## Conclusions

In this paper, an optical image encryption technique based on compressive sensing using fully optical means has been proposed. The simulations show that the method can be used to reconstruct the original image well with fewer measurements established by the Nyquist criterion and can be applied to gray-scale images and binary images to perform image encryption and compression in an all-optical system. With an optical image encryption technique and a compressive sensing technique, the method introduces an all-optical solution to sensing the original object, encrypting the object, and compressing the object in the analogue domain, which will present a superior scheme to overcome the limitations of the large holograms data volume for current optical image encryption systems. In addition, the DMD used in the setup of the compressive optical image encryption can perform high-speed measurements^27^; therefore, it is expected to be widely used for 3D object encryption, video secure transmission, real-time video encryption technology and future all-optical networks, such as real-time video security transmission and naked-eye 3D Television.

## Author Contributions

L.J. and L.J.S. conceived of the idea and wrote the main manuscript text. P.Y.Y. and L.R. prepared the figures. All authors reviewed the manuscript.

## Additional Information

**How to cite this article**: Li, J. *et al*. Compressive Optical Image Encryption. *Sci. Rep.* **5**, 10374; doi: 10.1038/srep10374 (2015).
